# Good vibrations: Sternal vibration enhances white matter density and interoceptive awareness

**DOI:** 10.21203/rs.3.rs-8329136/v1

**Published:** 2025-12-22

**Authors:** Negar Fani, Alexa Kondas, Vishwadeep Ahluwalia, Greg Siegle, Timothy McDermott, Alfonsina Guelfo, Travis Fulton, Aziz Elbasheir, Maya Karkare, Timothy Ely, Amanda Johnston, Rebecca Krawczak, Robert Krafty

**Affiliations:** Emory University School of Medicine; Emory University; Georgia Institute of Technology; University of Pittsburgh; Emory University; Emory University; Emory University; Emory University; Emory University; Emory University; Emory University; Emory University; Emory University

**Keywords:** diffusion-weighted imaging, interoception, neurostimulation, vibration, trauma

## Abstract

Vibration-based therapies are understudied but promising methods for alleviating psychiatric symptoms, particularly when paired with behavioral practices. The potential neuroplastic changes associated with a novel neurostimulation method, sternal vibration, and associations with clinical change remain unknown. We examined effects of sternal vibration paired with mindfulness meditation on change in white matter microstructure and dissociation using neurite orientation dispersion and density imaging (NODDI) in trauma-exposed adults with elevated dissociative symptoms. A total of 116 trauma-exposed adults with elevated dissociation completed MRI before/after eight mindfulness meditation sessions. Approximately half (n=60) received sternal vibration augmentation whereas n=56 had no augmentation. Self-reported dissociation was measured at each session with the Scale of Body Dissociation. Significant time-by-intervention type interactions showed vibration-specific improvements in body awareness (*p*=.032; *η*_*p*_^*2*^=.139) and increased neurite density index (NDI) in a region within the corticospinal tract (CST), the left cerebral peduncle (CP, *p*<.01, *η*_*p*_^*2*^=.102, *k*=15 voxels). This finding replicated in tractography analyses showing increased NDI in left (*p*=.007; *η*_*p*_^*2*^=.066) and right CST (*p*=.004; *η*_*p*_^*2*^=.075). Decreased body dissociation associated with increased CP NDI in those who received vibration (*p*=.011, *η*_*p*_^*2*^=.058); no associations between clinical and white matter change were observed with non-vibration interventions. Findings indicate that brief sternal vibration in the context of mindfulness meditation enhanced body awareness and neurite density in a tract of relevance to somatosensory integration. White matter changes corresponded with enhanced interoceptive awareness. Findings reveal the promise of sternal vibration as a low-cost, non-invasive neurostimulation method for enhancing interoception via neuroplastic alterations, with applications for various psychiatric populations.

## Introduction

Brief treatments and interventions that produce rapid and lasting relief for psychiatric disorders are urgently needed, particularly for posttraumatic stress disorder (PTSD)^1, 2^. Many trauma-exposed populations show high rates of attrition from, and limited responsiveness to, front-line exposure-based psychotherapies, i.e., prolonged exposure therapy^3–7^. Neurostimulation methods such as transcranial magnetic stimulation and transcutaneous vagal nerve stimulation, including vibrotactile vagal nerve stimulation, show promise for producing clinical change over a relatively short period of time^8^ and have broad appeal given their non-invasive nature. Corresponding neuroplastic changes produced by these methods may shift brain functional connectivity and structure in a way that accommodates environmental demands and enhances cognitive and emotional stability^9^.

When paired with behavioral interventions, such as meditative practices, neurostimulation may stabilize or improve emotional states, with interoceptive mechanisms—enhanced detection of body signals—being a potential mechanism of action^10–12^. Meditative interventions show emerging evidence of efficacy in PTSD^12–15^. Studies of meditative practices reveal neuroplastic changes that reflect normalized function and structure in neural circuits that engage during emotion regulation^16^. Regular meditators, as compared to novice or non-meditators, show differences in brain function^16^ and structure, including white matter changes, in regions involved with interoceptive and attentional processes, including the anterior cingulate cortex, insula and somatosensory cortices, although direction and strength of differences have been mixed across studies^17–20^. The non-invasive, non-pharmacological nature of meditative approaches have led to broad applications and widespread adoption in clinical populations, although difficulties with sustained engagement and drop out are frequently reported^21–24^. Neurostimulation, when paired with meditation, may enhance engagement and boost efficacy of these practices.

Vibration-based therapies, including whole body and focal vibration, are understudied but promising non-invasive neurostimulation methods that may be paired with meditation to enhance engagement and efficacy. These therapies show evidence for clinical and concomitant neuroplastic changes^25^. Heartbeat-like vibrations have been used to reduce acute stress and improve engagement and interoceptive awareness during meditation^26, 27^. Vibration stimulation at variable low frequencies (~ 10–120 Hz) has been shown to enhance nerve function in rehabilitative studies of rodents and humans with central and/or peripheral nerve injury, with some studies showing concurrent enhancements in cognitive and motor performance as well as mental state^28–33^. Although the mechanism of action remains unclear, vibration studies in rodents indicate restored density of synaptic terminals, neuronal proliferation, reduced microglial activation, axonal regeneration and remyelination^32^. The stimulation signal produced by vibration on skin mechanoreceptors travels through corticospinal pathways to affect cortical excitability, and potentially, neuroplasticity, in sensory/motor regions. Recently, vibrotactile stimulation to the cymba concha of the outer ear, used as a method of vagal nerve stimulation, produced working memory improvements in healthy adults^34^ and enhanced limbic connectivity in adults with epilepsy with implanted electrodes^35^. As such, vibration-based therapies have potential and myriad applications for treatment of psychiatric disorders.

When paired with meditation, vibration-based therapies are a form of neurostimulation that may facilitate therapeutic changes and potentiate neuroplastic effects and in psychiatric populations, introducing an exteroceptive point of focus that can facilitate sustained interoceptive awareness and meditative focus with minimal effort. In a randomized controlled trial for PTSD, we applied 100 Hz sternal respiratio-nsynced vibration with breath-focused meditation (BFM) during six 20-minute computer-guided sessions and found improvements in sustained attention and self-reported interoception^3^. In those who received vibration-augmented breath-focused mindfulness (VABF) as compared to BFM alone, interoceptive improvements corresponded with clinical changes, changes in autonomic regulation (high frequency heart-rate variability) and interoceptive network connectivity^36^. As such, the addition of sternal vibration to meditation appears to produce clinical effects that correspond with physiological changes.

Although these data and earlier studies of vibration-assisted therapies show the possibility of neuroplastic changes in response to vibration-augmented intervention, structural brain data on a larger scale would provide even more compelling evidence to support this claim.

Our ongoing multisite clinical trial is investigating potential changes in interoceptive network function in response to sternal vibration applied in two different ways during mindfulness meditation; we are using a 2 (vibration vs no vibration) by 2 (breath focus vs no breath focus) study design. Vibration conditions comprise VABF or pulsed vibration, non-vibration conditions comprise breath-focused mindfulness or open awareness mindfulness meditation. In this interim analysis, we examined potential effects of sternal vibration on white matter microstructure, given prior studies showing white matter plasticity with meditation^37, 38^. We used a diffusion MRI (dMRI) technique, neurite orientation dispersion and density imaging (NODDI), which provides a biophysical representation of tissue microstructure that involves estimation of a three-compartment model corresponding to regions of (i) intraneurites (axons, dendrites); (ii) extraneurites (soma, glia); and (iii) free water (cerebrospinal fluid)^39^. NODDI is a new modeling approach for diffusion-weighted data that may be more sensitive to changes in complex, non-Gaussian properties of white matter microstructure as compared to traditional modeling approaches (i.e., diffusion tensor imaging, DTI) and provides more granular details on white matter features with a high degree of replicability^40^. We examined change in neurite density index (NDI), which characterizes axonal and dendrite density; NDI differences have been consistently observed in psychiatric disorders vs controls^41^ and this index shows sensitivity to mild injury^42, 43^ and training effects over a short time^44^. We examined orientation dispersion index (ODI) as a secondary measure of white matter organization and integrity, which has shown sensitivity to mindfulness training^45^. Given the novelty of our intervention, we tested for potential white matter changes throughout the entire brain in a voxel-wise fashion. However, as our trial is designed to test the effects of vibration on interoceptive mechanisms, we expected changes in white matter tracts of relevance to interoception and multisensory integration^46–48^ and dissociation^49^, such as the corpus callosum, internal capsule, corona radiata, thalamic radiation, cingulum bundle, and corticospinal tract. To examine robustness of voxel-wise findings, we reconstructed all major white matter tracts using probabilistic tractography for replication analyses. Finally, we investigated self-reported changes in interoceptive awareness, examining vibration as a moderator of associations between change in interoceptive awareness and change in white matter microstructure.

## Methods

### Participants.

Data from 116 adults (*n*=88 female) aged 19–63 years (*M*_*age*_=31.30, *SD*=11.29) were included from an ongoing multi-site clinical trial for dissociation (NCT04670640), demographic characteristics detailed in Table 1, full medication/diagnostic characteristics provided in Table S1; there were no baseline differences for clinical or demographic characteristics between groups. Participants represent a subset of the clinical trial who completed post-intervention dMRI as of October 15th, 2024 and results represent interim analysis of non-primary outcomes. Individuals included in the trial met the following criteria: 1) experienced at least one DSM-5 PTSD Criterion A traumatic stressor, assessed via the Life Events Checklist for DSM-5 [LEC-5^50^]; 2) presence of clinically significant dissociation [score of >7 on the Multiscale Dissociation Index^51^ Depersonalization subscale]; 3)18–65 years of age. Exclusion criteria were: current severe suicidal ideation; acute psychosis or evidence of psychotic disorder; manic episode within the past year; severe substance use disorder; medical diagnosis affecting central nervous system function; history of moderate/severe traumatic brain injury; magnetic resonance imaging contraindications; non-correctable vision or hearing problems. Retention rates were marginally higher (*χ*^*2*^=2.97, *p*=0.085) for those who received vibration (96.6%) compared to those who did not (90.0%), with full details in Supplement (CONSORT diagram Figure S1). Research was conducted in accordance with the World Medical Association Declaration of Helsinki.

### Study Procedures

Following informed consent, participants completed a clinical interview visit to rule out any exclusionary conditions (e.g., schizophrenia). These clinical interviews included both the Clinician-Administered PTSD Scale for *DSM*-5 [CAPS-5^52^] and modules from the Mini Neuropsychiatric Interview [MINI^53^] administered by trained graduate-level or post-doctoral clinicians under the supervision of licensed psychologists. Eligible participants were scheduled to complete their first MRI visit (i.e., pre-intervention), then randomized to either a vibration or non-vibration intervention condition, each of which included eight visits, during which they completed a body connectedness measure. Covariate adaptive randomization^54^ based on biological sex assigned at birth, race, ethnicity, age group, and baseline dissociation severity was used for randomization, detailed in Supplement. After eight intervention visits, participants completed a post-intervention MRI scan and clinical interview.

### Intervention Procedures.

Participants were randomized to one of 4 conditions that were either a vibration (VABF, pulsed vibration) or non-vibration (breath-focused mindfulness, open awareness) intervention. Participants remained in the same intervention and received the same instructions for all eight visits. During each visit, participants received brief instructions related to their respective intervention condition via computer monitor. Irrespective of the randomized intervention condition, participants wore a low frequency haptic transducer on the sternum and a pneumatic respiration cushion to measure respiration (Figure S2). Intervention visits consisted of six, three-minute blocks of mindfulness meditation, consistent with the assigned intervention’s instructions (18 min total). Vibration from the transducer lasted ~6 seconds [synchronized to the start of exhalation in VABF and triggered every 6 seconds in pulsed vibration]. Full details on the device and interventions are provided in Supplement.

### Body Dissociation.

The Scale of Bodily Connectedness (SBC) is a 20-item Likert-style self-report questionnaire assaying body/interoceptive awareness and dissociation.^55^. Responses range from 1 (“not at all true”) to 5 (“very true”), and higher scores indicating greater perceived bodily connectedness. After each intervention session participants responded to their perceived connection to their physical body during the session, including facets of body awareness and body dissociation. Averaged scores were used in analysis.

### MRI Acquisition and Diffusion-weighted Image Processing.

MRI scans were acquired on two research-dedicated Siemens 3-Tesla Prisma^fit^ MRI systems, one per site. Multi-shell diffusion weighted imaging (DWI) was obtained with optimal angular coverage using 128 diffusion directions distributed over 4 shells. 3D-T1 MPRAGE with 0.8mm^3^ isotropic resolution was acquired for co-registration. Diffusion-weighted image processing and analysis were conducted using FMRIB Software Library [FSL version 4.1^56^]; multiple compartment modeling performed on diffusion-weighted data using NODDI^57, 58^ to generate Neurite Density Index (NDI) and Orientation Dispersion Index (ODI) maps. Quality checks were performed by calculating the temporal signal-to-noise ratio (tSNR) across each diffusion volume. To account for differences in scanner and scan quality via tSNR, scalar maps were harmonized using ComBat-GAM/neuroHarmonize^59^. Full image processing details provided in Supplement.

### Voxel-wise Metrics and Probabilistic Tractography.

Voxel-wise differences in NODDI scalar indices were assessed using Tract-based Spatial Statistics (TBSS, version 1.2, in FSL)^60^. Tracts that demonstrated significant time and/or intervention-related changes in primary voxel-wise analyses were reconstructed using probabilistic tractography to assess for replication. We used XTRACT in FSL^61–63^ to reconstruct 42 major white matter tracts using pre-defined, anatomically-constrained seed, target, exclusion, and stop masks in participant’s diffusion space. Mean NODDI metrics were extracted from tracts of interest. Full details provided in Supplement.

### Statistical Analyses.

We first examined potential voxel-wise group differences in ODI and NDI maps using nonparametric permutation testing implemented in FSL (*randomize*). A primary 2×2 ANOVA examined main effects of time (pre/post-intervention), condition (vibration vs. no vibration), and their interaction. Permutation testing was performed using 5000 permutations to ensure robust statistical inference. Given the novelty of our intervention method and unknown effects, as well as known limitations of traditional Type I error correction methods in detecting subtle effects—particularly in smaller samples or in novel imaging metrics—an uncorrected cluster-based threshold of *p*<0.01 and minimum cluster extent of 10 contiguous voxels was applied, consistent with commonly-used exploratory thresholds^64^. Voxel-wise MRI analyses in novel (and in-progress) studies such as ours may reveal small but meaningful targets for future investigations, which more stringent thresholds may obscure; as such, we used prior guidance on prioritizing management Type II error in such studies to determine our threshold for initial voxel-wise analyses^65,66^. To affirm robustness of findings, probabilistic tractography was used to determine whether results replicated while testing for the inclusion of additional covariates using Bayesian information criterion (BIC) to determine model of best fit. We collapsed vibration conditions into one group and non-vibration conditions into another to preserve statistical power in analyses, however, we also tested effects across all four intervention conditions separately to assess for any differentiation between the four conditions. Where voxel-wise significant results were observed, mean ODI and NDI values were extracted from these clusters to determine associations with clinical change; we examined change in self-reported body connectedness (SBC Body Awareness/Body Dissociation subscales) using multiple linear regression with vibration as a moderator of the relationship between change in self-reported body connectedness and white matter microstructure using PROCESS Macro in SPSS Version 29.0^67^. To ensure specificity of findings to dissociation, we ran additional covariate tests with medication use and PTSD symptoms as covariates.

## Results

### Voxel-wise Effects of Vibration on NDI and ODI.

**Figure 1:**
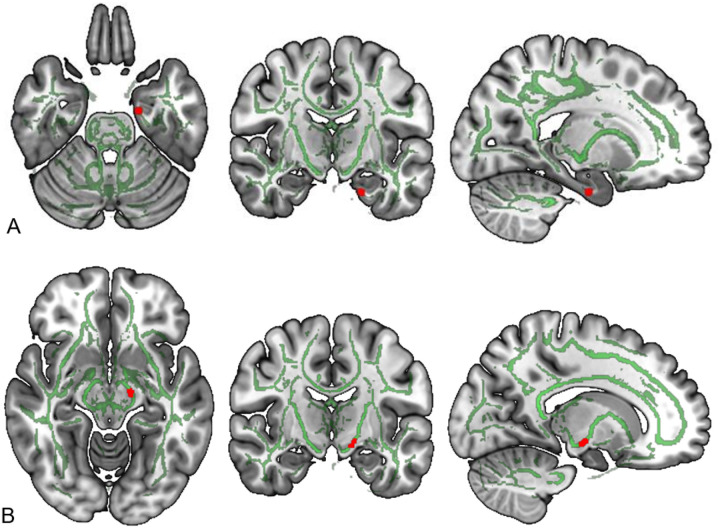
Voxelwisechanges in neurite density index (NDI), significant findings highlighted in red, skeletonized white matter tracts in green. A. Increased parahippocampalcingulum NDI observed from pre- to post-intervention. B) Time-by-vibration interaction indicating increased NDI in left cerebral peduncle in vibration conditions.

Using TBSS, we first conducted an ANOVA on NDI testing for main effects of time, vibration, and a time-by-vibration interaction. A main effect of time was observed with NDI in a cluster within the right parahippocampal cingulum (peak voxel MNI coordinates: MNI x=111, y=114, z=43; k=12; *p*=.001, *η*_*p*_^*2*^=.109; Figure 1a). Explication of this interaction revealed increased NDI over time in both vibration and non-vibration groups (Figure 1a). A significant time-by-vibration interaction on NDI was also observed in a voxel cluster within the left cerebral peduncle (Figure 1b; peak voxel MNI coordinates: x=106, y=113, z=63; k=15; *p*=.001; *η*_*p*_^*2*^=.102); explication of this interaction revealed increased NDI over time in the vibration group as compared to the non-vibration group. For ODI, there were no significant main effects of time, vibration, or a time-by-vibration interaction.

### Replication Analyses with Tractography.

**Figure 2:**
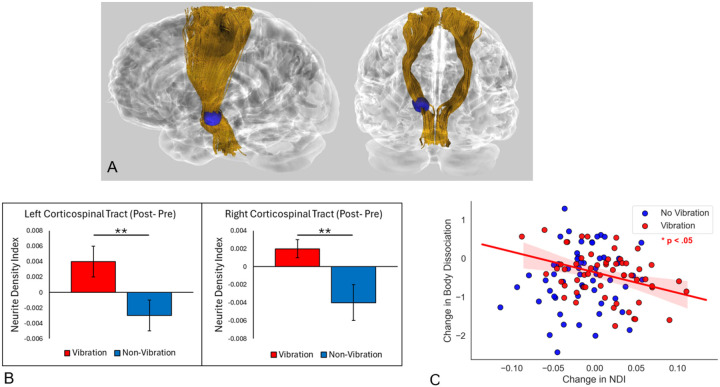
**A.** Tractographicreconstruction of corticospinal tract (gold), purple sphere indicating position of voxelwisetime-by-condition findings (cerebral peduncle). **B.** Increased corticospinal tract neurite density index (NDI) observed in left (p=.007; ηp2=.066) and right hemisphere (p=.004; ηp2 =.075) in vibration conditions. **C**) Moderating effect of vibration on white matter and self-reported body dissociation. Increased NDI associated with decreased body dissociation in vibration group (β=−5.33, p=.013) but no significant relationship between NDI change and body dissociation change was observed in the non-vibration group (β=3.25, p=.20).

To assess whether observed effects of time and vibration on NDI replicated with a different method, we used probabilistic tractography in the subject’s diffusion space to reconstruct the corticospinal tract, CST, which includes the cerebral peduncle, and parahippocampal cingulum (left and right temporal sections of the cingulum) and extracted NDI averages, which were then used in 2 × 2 ANOVAs while testing for the inclusion of additional covariates with BIC. These analyses were conducted bilaterally to be comprehensive. Results showed significant time-by-vibration interactions for both the left (*p*=.007; *η*_*p*_^*2*^=.066) and right (*p*=.004; *η*_*p*_^*2*^=.075) CST (Figure 2a), both of which showed increased NDI in the vibration group over time, whereas the non-vibration group showed non-significant change over time (Figure 2c). These models did not include any additional covariates based on the results of the BIC model comparison, but confirmatory ANOVAs including covariates of age and biological sex still showed significant time-by-vibration interaction effects for both the left (*p*=.006; *η*_*p*_^*2*^=.069) and right CST (*p*=.004; *η*_*p*_^*2*^=.076; Figure 2a,b). Age and biological sex were included as covariates in confirmatory ANOVAs as these covariates constituted the next best models in BIC after the models with no covariates, and prior literature has extensively documented the influence of age and biological sex on white matter microstructure^68^. ANOVAs on the left and right temporal section of the cingulum were not significant (*p*’s>.10). Additionally, ANOVAs testing these effects between all four intervention conditions showed significant time-by-condition interactions for both the left (*p*=.044; *η*_*p*_^*2*^=.073) and right (*p*=.029; *η*_*p*_^*2*^=.081) CST. However, these results were comparable to those in primary models with VABF and V showing similar increases in NDI across time, unlike the BF and OA groups. Full results for these replication analyses are provided in Table S2.

### Self-reported Changes in Body Connectedness.

Results of a repeated-measures ANOVA on SBC: Body Awareness showed a significant main effect of time (*p*<.001; *η*_*p*_^*2*^=.310) and a significant time-by-vibration interaction (*p*=.032; *η*_*p*_^*2*^=.139). The magnitude of increase in self-reported body awareness was greater for the vibration vs non-vibration group across the intervention sessions. There were significant pairwise differences at session 5 (*p*=.003) and session 6 (*p*=.023). The ANOVA testing these same effects on SBC: Body Dissociation showed a significant main effect of time (*p*<.001; *η*_*p*_^*2*^=.416) but no time-by-vibration interaction (*p*=.47). This main effect of time was a reduction in self-reported body dissociation across sessions that was equivalent across the vibration and non-vibration groups. Full results for these analyses are provided in Table S3.

### White Matter Change and Change in Body Connectedness: Moderation by Vibration Condition.

Moderation analysis examining moderating effects of vibration on cerebral peduncle NDI change and change in self-reported interoception (i.e., SBC subscales) showed a significant moderating effect of vibration on the association between NDI in the left cerebral peduncle and SBC: Body Dissociation (*β*=−8.58; *p*=.011, *η*_*p*_^*2*^=.058). This moderating effect was such that increased NDI was associated with decreased body dissociation in those who received a vibration-augmented intervention (*β*=−5.33, *p*=.013), whereas no significant relationship between NDI change and body dissociation change was observed in the non-vibration group (*β*=3.25, *p*=.20; Figure 2c). This moderating effect remained significant when covarying changes in medication use or PTSD symptoms (*β*=−7.86; *p*=.046, *η*_*p*_^*2*^=.064). Further, the same pattern was observed when testing these relationships across all four intervention conditions separately (Figure S3). The regression model testing these associations between left cerebral peduncle NDI and changes in SBC: Body Awareness was not significant, nor were models testing associations between changes in parahippocampal cingulum cluster values and changes in SBC subscales.

## Discussion

We examined the effects of brief sternal vibration (pulsed or respiration-synced) on change in white matter, assessed via a novel and sensitive white matter modeling method (NODDI) and how these changes corresponded with clinical change. Vibration-specific improvements were observed over time with self-reported interoception. Voxel-wise analyses showed pre- to post-intervention changes in neurite density/NDI in the parahippocampal cingulum, a region of relevance to numerous cognitive and emotional processes. However, vibration produced specific NDI increases from pre- to post-intervention —with large effect size—in a tract essential to somatosensory integration, the corticospinal tract, specifically within the cerebral peduncle. Replication with a different analytic method, probabilistic tractography, showed similar, specific vibration-related NDI enhancement in the corticospinal tract, with a moderate effect size. Further, participants who received vibration showed increased cerebral peduncle NDI that associated with decreased self-reported body dissociation, a relationship that was not observed in the non-vibration meditation group. To our knowledge, this is the first study to show that vibration-augmented meditation enhances white matter integrity, with specific effects on a pathway of relevance to interoception and somatosensory processes. These data illuminate the promise of brief, vibration-augmented therapies on interoceptive network plasticity for populations with trauma and stress-related disorders.

Main effects of time (pre- to post-intervention change) were observed in the cingulum bundle, particularly within the temporal section which extends from the parahippocampal region to the prefrontal cortex. Changes in this pathway have been observed with other types of therapies and cingulum regions have been successfully targeted in deep brain neurostimulation therapies for PTSD^69–72^ to enhance mood and reduce anxiety; cingulum changes have also been observed following meditation^38^. Our findings of increased NDI in this pathway may indicate non-specific effects of behavioral interventions on cingulum plasticity. However, there were no associations of change in this tract with clinical changes, complicating interpretation of findings.

In contrast, vibration-specific changes in NDI were observed in the CST, particularly within the cerebral peduncle, and these white matter changes associated with clinical changes—decreased body dissociation—only in those who received vibration. The CST is a white matter pathway that originates in the primary somatosensory cortex and passes through the posterior limb of the internal capsule and midbrain before entering the spinal column; among white matter pathways, it may be most central to somatosensory integration and the modulation of incoming sensory information^47, 73^. The cerebral peduncle serves as a connection between the midbrain and the thalamus, and is essential to sensory learning processes, with some evidence for its role in sensory integration and attention to sensory/interoceptive cues^74, 75^. Vibration moderated associations between CST/cerebral peduncle white matter and clinical/self-reported interoception changes; in participants who received vibration, increased NDI in this region associated with increased interoceptive awareness (decreased body dissociation), a relationship that was not observed in non-vibration conditions. Enhanced white matter microstructure of the CST has been observed following some somatically oriented meditative interventions, such as Tai Chi^76^, and enhanced neuronal density in gray matter has been observed in cortical, limbic and cerebellar regions following mindfulness meditation more specifically^77^. Given that NDI indexes the density of axons and dendrites, it is possible that the addition of vibration to meditation produces greater plasticity of CST white matter. Prior studies using cognitive and motor learning tasks have shown similar changes in NODDI metrics^44, 78, 79^, suggesting the value of these white matter metrics in assessing network remodeling following beneficial mental and/or behavioral practices.

Invasive and non-invasive vagal nerve stimulation (magnetic or electrical) shows similar effects on white matter pathways, and may shed light on potential mechanisms of change. Pre-clinical and human studies of neurological injury (e.g., demyelinating disorders, stroke) show that VNS paired with motor tasks/rehabilitation training produces greater CST plasticity/white matter myelination and recovery following the paired intervention^80, 81^, with recent studies suggesting oligodendrocyte generation and diminished neuroinflammation with these therapies^82, 83^. Vagal nerve stimulation when paired with rehabilitation techniques is thought to enhance recovery by promoting learning through enhanced neuroplasticity^84^. Given the placement of the vibrating element on the sternum (and relative proximity to thoracic branch of vagus nerve), it is possible that rhythmic sternal vibration may, in part, exert its effects through a similar mechanism. Vibration stimulation, which has shown effects on emotion-related and somatosensory regions^85^, may enhance attention to sensory stimuli, including (but not limited to) the breath, serving as “training wheels” for interoceptive treatments. In combination, vibration may serve to enhance interoceptive focus via changes in interoceptive/sensory learning pathways, improving engagement and leading to more potent clinical changes. Although the long-term persistence of these changes is a topic of future study, these data provide promising evidence that this combined training approach produces neuroplastic and clinical changes within a short (<2 month) period of time, without the need for highly specialized professionals.

We acknowledge several limitations to this study, including the fact that, in the absence of direct observations (e.g., pre-clinical experimental data, in-vivo electroencephalographic recording), the previously-discussed mechanisms of white matter change are speculative. Further applications of both closed-loop and pulsed sternal vibration and longer-term evaluation are needed to examine in-vivo mechanistic changes and the extent and duration of associations with white matter and clinical changes. Use of other interoceptive assessment methods (e.g., respiratory sensitivity task) would also add depth to our understanding of how sternal vibration affects different facets of interoception. We also did not observe significant differences between the vibration sub-groups, which could reflect insufficient statistical power to detect potential differences. Nonetheless, this study is the first of its kind to show how short-term sternal vibration accompanied by meditation may produce neuroplastic changes that correspond with clinical changes, with further outcomes to be reported at the end of this clinical trial.

In conclusion, the findings from this study of diverse trauma-exposed individuals with PTSD symptoms show that 8 sessions of brief sternal vibration in the context of mindfulness meditation produces changes in white matter (neurite density) that correspond with enhanced interoceptive awareness, assessed via self-report ratings. Interoceptive disruptions are common in trauma survivors with traumatic stress-related symptoms, inclusive of body disconnection/dissociation^86^. In this study, we use a somatically-focused neurostimulation method to enhance interoceptive functions, with a focus on body awareness; sternal vibration appeared to enhance these functions via neuroplastic changes of an interoceptive pathway, the CST. Notably, high completion rates of vibration interventions indicate that this type of exteroceptive stimulation enhanced intervention engagement, which is challenging for novice meditators and populations with mental health disorders more generally. Given the brevity, low-cost, and non-invasive nature of this intervention, these data show promising evidence for this type of neurostimulation as an effective intervention method, with broad applications for mental health disorders.

## Supplementary Material

Supplementary Files

This is a list of supplementary files associated with this preprint. Click to download.

- MINDNODDISupplement.docx

## Figures and Tables

**Table 1.** Demographic Characteristics (N=116)

Mindfulness Training Group:	Vibration (*n*=60)	Non-Vibration (*n*=56)	
**Site**	**% (*n*)**		**Pearson χ** ^**2**^ **=2.1**
Emory University	63.3 (38)	50.0 (28)	
University of Pittsburgh	36.7 (22)	50.0 (28)	
**Sex Assigned at Birth**	**% (*n*)**		**Pearson χ** ^**2**^ **=0.04**
Female	76.7 (46)	75.0 (42)	
Male	36.7 (14)	25.0 (14)	
**Race**	**% (*n*)**		**Pearson χ** ^**2**^ **=3.5**
White	43.3 (26)	50.0 (28)	
Black or African American	40.0 (24)	35.7 (20)	
Asian	5.0 (3)	5.4 (3)	
Multiracial	11.7 (7)	8.9 (5)	
**Ethnicity**	**% (*n*)**		**Pearson χ** ^**2**^ **=0.02**
Hispanic/Latinx	13.3 (8)	12.5 (7)	
Not Hispanic/Latinx	86.7 (52)	87.5 (49)	
**Education Level**	**% (*n*)**		**Pearson χ** ^**2**^ **=3.3**
High school diploma or GED	15.0 (9)	7.1 (4)	
Some college (at least 1 year)	33.3 (20)	33.9 (19)	
Technical school or Associate’s	5.0 (3)	10.7 (6)	
College diploma	23.3 (14)	28.6 (16)	
Graduate or professional degree	23.3 (14)	19.6 (11)	
**Monthly Income**	**% (*n*)**		**Pearson χ** ^**2**^ **=5.8**
$0-$249	15.0 (9)	5.4 (3)	
$250-$499	5.0 (3)	7.1 (4)	
$500-$999	6.7 (4)	14.3 (8)	
$1,000-$1,999	18.3 (11)	26.8 (15)	
$2,000 or more	55.0 (33)	46.4 (26)	
	**M (*SD*, Range)**		**Independent *t* test**
**Age (years)**	30.90 (11.30, 18–61)	28.46 (11.63, 18–61)	*t*=1.1
**Days Between Scans**	47.48 (27.22, 20–163)	50.07 (18.84, 21–97)	*t*=-0.6
**LEC Experienced Total**	3.23 (2.25, 0–9)	3.86 (2.30, 0–12)	*t*=-1.5
**LEC Witnessed Total**	2.15 (2.08, 0–10)	2.29 (2.36, 0–9)	*t*=-0.3

LEC=Life Events Checklist
